# Accuracy in HIV Rapid Testing among Laboratory and Non-laboratory Personnel in Zambia: Observations from the National HIV Proficiency Testing System

**DOI:** 10.1371/journal.pone.0146700

**Published:** 2016-01-08

**Authors:** Sheila Mwangala, Kunda G. Musonda, Mwaka Monze, Katoba K. Musukwa, Knut Fylkesnes

**Affiliations:** 1 Virology Laboratory, Department of Pathology and Microbiology, University Teaching Hospital, Lusaka, Zambia; 2 Centre for International Health, Department of Global Public Health and Primary Care, University of Bergen, Bergen, Norway; 3 Pathogen Molecular Biology Department, London school of Hygiene and Tropical Medicine, University of London, London, United Kingdom; Rutgers University, UNITED STATES

## Abstract

**Background:**

Despite rapid task-shifting and scale-up of HIV testing services in high HIV prevalence countries, studies evaluating accuracy remain limited. This study aimed to assess overall accuracy level and factors associated with accuracy in HIV rapid testing in Zambia.

**Methods:**

Accuracy was investigated among rural and urban HIV testing sites participating in two annual national HIV proficiency testing (PT) exercises conducted in 2009 (n = 282 sites) and 2010 (n = 488 sites). Testers included lay counselors, nurses, laboratory personnel and others. PT panels of five dry tube specimens (DTS) were issued to testing sites by the national reference laboratory (NRL). Site accuracy level was assessed by comparison of reported results to the expected results. Non-parametric rank tests and multiple linear regression models were used to assess variation in accuracy between PT cycles and between tester groups, and to examine factors associated with accuracy respectively.

**Results:**

Overall accuracy level was 93.1% (95% CI: 91.2–94.9) in 2009 and 96.9% (95% CI: 96.1–97.8) in 2010. Differences in accuracy were seen between the tester groups in 2009 with laboratory personnel being more accurate than non-laboratory personnel, while in 2010 no differences were seen. In both PT exercises, lay counselors and nurses had more difficulties interpreting results, with more occurrences of false-negative, false-positive and indeterminate results. Having received the standard HIV rapid testing training and adherence to the national HIV testing algorithm were positively associated with accuracy.

**Conclusion:**

The study showed an improvement in tester group and overall accuracy from the first PT exercise to the next. Average number of incorrect test results per 1000 tests performed was reduced from 69 to 31. Further improvement is needed, however, and the national HIV proficiency testing system seems to be an important tool in this regard, which should be continued and needs to be urgently strengthened.

## Background

HIV/AIDS is still one of the world’s most devastating pandemics, with sub-Sahara Africa the most affected region [1,2]. HIV testing remains a critical entry point for prevention, treatment and care. Since the mid 1980’s when an accurate and reliable antibody test became available, HIV testing has been offered mainly through client-initiated voluntary counseling and testing (VCT) services [3,4]. Uptake has been very low despite high willingness to be tested [5,6] and stigma has been identified as one of the strongest barriers to VCT [7]. As antiretroviral treatment (ART) became available in low-income settings, Provider Initiated Testing and Counseling (PITC) or Routine opt-out Testing and Counseling (RTC) has been recommended to be implemented in countries with generalized epidemics. Despite a steady increase in test rates over the years [8,9], uptake is still unacceptably low and distributed in an inequitable way [10].

To meet the increasing need for HIV testing, testing methods have shifted from sophisticated techniques such as enzyme linked immunosorbent assays (ELISAs), which are usually performed in a traditional laboratory by highly trained laboratory professionals, to rapid tests (RTs) [11,12]. RTs are simple to perform, are accurate, give results without the need for laboratory equipment and have allowed provision of test results and post-test counseling in a single visit [13–16]. This has allowed non-laboratory personnel such as lay counselors and nurses to perform HIV tests [11–13], thus in keeping with the WHO recommendation of task-shifting of HIV testing services [17,18]. Concerns have been raised, however, on the effects of this expansion on quality of testing and accuracy of test results [12,13]. Given the high volume of testing, even a small error rate can result in a high number of misdiagnosed cases. For example a 5% error rate in testing 2 million people could result in 100,000 erroneous diagnoses. This calls for coherent quality assurance (QA) systems to regulate and monitor performance of HIV testing [11,12,19].

Countries such as Uganda, Zimbabwe, China and India have reported the establishment of national QA programs through national reference laboratories (NRLs). These programs assess quality of testing and ensure accuracy and reliability of test results [11,20–22]. QA monitors all aspects of the testing process (pre-analytic, analytic and post-analytic) and is implemented in mainly two ways: 1) Internal Quality Control (IQC) and 2) External Quality Assessment (EQA). IQC is used to evaluate and ensure that the test precision is optimal, while EQA provides additional external checks to the testing process [23–25]. EQA is usually implemented through a combination of 3 approaches: 1) Re-testing 2) Site supervisory visits (SSV) 3) Proficiency testing (PT). Due to the huge volume of testing required, re-testing has proved to be neither practical nor achievable where human and financial resources are limited [12]. SSVs, which involve an on-site review of all aspects of the quality system, require a large pool of trained personnel to conduct costly visits. PT, a generally effective EQA approach [12,25], is a tool where simulated specimens issued by the NRL are tested by participating HIV testing sites and individual site performance is assessed by comparison to the NRL expected results and collective performance of all participants [26]. PT establishes the degree of accuracy of test results, evaluates technical competence and identifies staff training needs [13,19,27].

Though HIV RTs are simple to perform, inadequate training and experience of test performers may present a limitation to achieving accurate diagnostic testing [11,14,19]. Test kit manufacturer’s instructions often only illustrate examples of clear-cut results along with strong-intensity test and control lines. Thus operators without in-depth training and experience may have difficulties in interpreting weakly reactive results, or in understanding and resolving indeterminate (inconclusive or discrepant) [14,19] or invalid (un-interpretable) results. Some studies have suggested that difficulties of this nature may occur more among non-laboratory personnel if not properly trained [28,29]. Comprehensive hands-on training and on-going performance monitoring has been suggested to be essential for all test performers [12,14,30]. In addition to training, other factors such as adherence to testing procedures, policy development, test kit availability, supervision, skilled human resource availability and workload need to be addressed to ensure quality of testing and the successful expansion of the service [11].

Adult HIV prevalence in Zambia has been estimated at 13.5% [2]. An overall decline in HIV incidence has been observed since the mid-1990s but with marked geographical differences [31,32,33] and has been positively associated with education attainment [34,35]. HIV testing services have been available in Zambia since the late 1980s and with a country-wide scale up of VCT from 1998. In recent years, VCT services have expanded from about 650 sites in 2006 to about 1689 sites in 2010 [36]. With this expansion, a sharp rise in reporting ever tested for HIV has been observed [37,38]. The Ministry of Health (MOH) and its collaborating partners provide HIV rapid testing training to prospective testers of various professional backgrounds [39]. Through the University Teaching Hospital Virology Laboratory (UTHVL), the NRL, the MOH established the Zambia National Quality Assurance Program (ZANQAP) in 2009, with the aim of monitoring the performance of HIV testing across the country. PT and SSVs are the two main EQA approaches being practiced [40]. Despite the rapid scale up of HIV testing services in high HIV prevalence countries, documentations in the literature assessing accuracy of test results are few. The aim of this study was to assess the overall level of accuracy in HIV rapid testing in Zambia and variation by different groups of testing personnel and to examine factors that are associated with accuracy.

## Methods

### Site selection and participation

The data stem from two annual national PT exercises that were conducted in 2009 and 2010 in selected rural and urban HIV testing sites across the nine provinces of Zambia. The first PT cycle (PT1) in 2009 targeted as many sites as could possibly be reached. Officials from the provincial and district health offices assisted in the identification of HIV testing sites for participation. In 2010 (PT2), MOH partners that technically and financially support various sites across the country were involved in site selection. The sites were selected to represent all types and levels of facilities in the country. The sites included those in public health facilities (referral hospitals, provincial hospitals, district hospitals, military hospitals, mine hospitals, health centres and health posts), mission hospitals and private hospitals/clinics (stand alone, company or project). The number of targeted sites in PT1 was 550 and was increased to 680 in PT2.

### Proficiency testing

The current HIV PT implemented involves the use of dried tube specimens (DTS), which has been adopted to overcome the rigorous storage and transportation conditions required with the use of liquid serum and plasma specimens for such an exercise. DTS are safe, easy to generate, are stable for at least one month within a broad temperature range during storage and transport and hence do not require cold chain maintenance. In both PT rounds, commercially sourced serum panels (ZeptoMetrix, Buffalo NY) of known HIV status were conditioned at the NRL into DTS using the protocol developed by Parekh et al [41], briefly as follows: 20ul of serum pre-mixed with 0.1% (v/v) green dye were transferred into 2ml microcentrifuge tubes. The tubes were left open to dry overnight at room temperature in a laminar flow hood. The next day the tubes were recapped in readiness for packaging and distribution to sites. Once dried, the DTS are rehydrated (reconstituted) with PT buffer [12,21,41].

For each PT, the PT panel consisted of five blinded DTS specimens (two negative and three positive including one weakly positive). After re-testing of the DTS for quality control i.e. testing of 10% of the specimens by different testers for consistency, PT packages for each site were prepared. Each PT package included the five DTS, one 1.5ml vial of PT buffer, a transfer pipette, instructions for reconstitution and reporting of results and a self-administered questionnaire to capture demographic and HIV rapid testing information of the sites [12,41].

Distribution of the PT packages to the sites and return of results to the NRL was done by the district health offices in PT1 and by the national courier service and MOH partners in PT2. The sites were instructed to reconstitute the DTS upon receipt and to test them in the same way they test routine client samples, following the national HIV rapid testing algorithm. The Zambia national HIV rapid testing algorithm is based on serial testing, which specifies the use of two rapid antibody assays, Determine^®^ HIV-1/2 (Abbott Laboratories, Abbott Park, IL) as a screening test and if reactive, Uni-Gold^™^ HIV (Trinity Biotech Plc, Wicklow, Ireland) as a confirmatory test. At the time of the PT exercises, the testing algorithm included a third test, SD Bioline HIV-1/2 (Standard diagnostics Inc., Kyonggi-do, Korea) as a tie-breaker [25]. One tester tested the DTS per site. The sites were instructed to complete testing, report and send results together with the filled in questionnaire to the NRL within 1 month of receipt of the PT panels. Performance reports were compiled and sent to the individual sites. Results for all the sites were compiled to give the overall accuracy level for each PT cycle. Supervisory visits to sites that scored less than 100% were made by the NRL technical team in both PT1 and PT2. The sites were assessed e.g. for availability of test kits and proper test performance and received technical assistance on testing.

### Data analysis

Data was entered using EpiData 3.1 and MS excel 2007 and analyzed using IBM SPSS version 20 for windows for overall and trends analysis.

Tester profession was categorized into four groups as: lay counselors, nurses, laboratory personnel (scientists, technologists and assistants) and others (microscopists, Environmental Health Technologists, clinical officers and doctors). Responses to having received HIV rapid testing training was categorized into four groups as seen in Table 1. HIV testing work experience was dichotomized as ‘less’ or ‘more’ than one year. The number of staff performing HIV testing at a site was categorized into three groups as 0–5, 6–10 and 11 or more. Responses to if IQC is performed at the site was dichotomized as ‘yes’ and ‘no’. Adherence to the national HIV testing algorithm during the PT cycles was dichotomized as ‘yes’ and ‘no’.

**Table 1:** General background and proficiency testing characteristics by tester profession.

Characteristics	Lay counselors		Nurses		Laboratory personnel		Others		Total	
	PT1	PT2	PT1	PT2	PT1	PT2	PT1	PT2	PT1	PT2
	n (%)	n (%)	n (%)	n (%)	n (%)	n (%)	n (%)	n (%)	n (%)	n (%)
**Distribution of testers**	101 (36.1)	185 (38.8)	117 (41.8)	160 (33.5)	46 (16.4)	106 (22.2)	16 (5.7)	26 (5.5)	282 (100)	488 (100)
**Type of Institution**										
Public	92 (91.1)	144 (86.2)	112 (95.7)	116 (78.9)	41 (89.1)	63 (62.4)	14 (87.5)	22 (88.0)	260 (92.2)	355 (78.7)
Faith based	7 (6.9)	17 (10.2)	4 (3.4)	17 (11.6)	4 (8.7)	19 (18.8)	2 (12.5)	0 (0.0)	18 (6.4)	54 (12.0)
Private	2 (2.0)	6 (3.6)	1 (0.9)	14 (9.5)	1 (2.2)	19 (18.8)	0 (0.0)	3 (12.8)	4 (1.4)	42 (9.3)
**Service offered**										
VCT	51 (50.5)	51 (28.5)	23 (19.7)	18 (11.8)	1 (2.2)	5 (5.0)	2 (12.5)	3 (11.5)	78 (27.7)	78 (16.7)
DCT + TB	3 (3.0)	12 (6.7)	20 (17.1)	10 (6.6)	4 (8.7)	1 (1.0)	2 (12.5)	3 (11.5)	29 (10.3)	26 (5.6)
PMTCT	11 (10.9)	12 (6.7)	20 (17.1)	19 (12.5)	1 (2.2)	4 (4.0)	0 (0.0)	0 (0.0)	32 (11.3)	35 (7.5)
LAB	1 (1.0)	1 (0.6)	1 (0.9)	0 (0.0)	31 (67.4)	34 (34.0)	3 (18.8)	0 (0.0)	36 (12.8)	36 (7.7)
More than 1 or all	35 (34.7)	103 (57.5)	53 (45.3)	105 (69.1)	9 (19.6)	56 (56.0)	9 (56.3)	20 (76.9)	107 (37.9)	291 (62.4)
**Location of site**										
Rural	63 (62.4)	74 (40.0)	43 (36.8)	39 (24.4)	12 (26.1)	29 (27.4)	15 (93.8)	16 (61.5)	135 (47.9)	162 (33.2)
Urban	38 (37.6)	111 (60.0)	74 (63.2)	121 (75.6)	34 (73.9)	77 (72.6)	1 (6.2)	10 (38.5)	147 (52.1)	326 (66.8)
Training*										
HIV Rapid Testing		118 (66.7)		81 (55.1)		35 (74.5)		12 (54.5)		248 (62.3)
PMTCT		11 (6.2)		32 (21.8)		3 (6.4)		4 (18.2)		51 (12.8)
Psychosocial counseling		43 (24.3)		30 (20.4)		2 (4.3)		3 (13.6)		79 (19.8)
Other/no training		5 (2.8)		4 (2.7)		7 (14.9)		3 (13.6)		20 (5.0)
**Time since last training**										
< one year ago	44 (51.2)	61 (36.7)	34 (31.2)	37 (26.8)	8 (32.8)	13 (32.5)	6 (46.2)	7 (33.3)	93 (39.6)	119 (32.3)
> one year ago	42 (48.8)	105 (63.3)	75 (68.8)	101 (73.2)	17 (68.0)	27 (67.5)	7 (53.8)	14 (66.7)	142 (60.4)	249 (67.7)
**Visited by trainer**										
Yes	73 (79.3)	145 (82.9)	66 (58.9)	93 (64.1)	13 (54.2)	32 (65.3)	5 (41.5)	15 (62.5)	158 (65.3)	289 (72.6)
No	19 (20.7)	30 (17.1)	46 (41.1)	52 (35.9)	11 (45.8)	17 (34.7)	7 (58.3)	9 (37.5)	84 (34.7)	109 (27.4)
**Work experience**										
< one year	53 (55.8)	29 (15.9)	20 (18.2)	17 (10.8)	4 (12.1)	10 (11.6)	4 (28.6)	3 (12.5)	81 (31.9)	61 (13.3)
> one year	42 (44.2)	153 (84.1)	90 (81.8)	140 (89.2)	29 (87.9)	76 (88.4)	10 (71.4)	21 (87.5)	173 (68.1)	396 (86.7)
**Perform IQC**										
Yes	74 (97.9)	121 (69.9)	108 (98.2)	71 (49.0)	40 (87.0)	82 (83.7)	14 (87.5)	13 (68.4)	238 (97.9)	293 (66.3)
No	3 (3.9)	52 (30.1)	2 (1.8)	74 (51.0)	6 (13.0)	16 (16.3)	2 (12.5)	6 (31.6)	5 (2.1)	149 (33.7)
DTS Reconstitution*										
Easy		133 (84.7)		132 (95.0)		103 (100)		20 (83.3)		395 (91.4)
Difficult		3 (1.9)		3 (2.2)		0 (0.0)		4 (16.7)		10 (2.3)
Unable		21 (13.4)		4 (2.9)		0 (0.0)		0 (0.0)		27 (6.3)
**Timer available**										
Yes	29 (33.7)	46 (26.3)	19 (17.9)	47 (31.3)	19 (48.7)	54 (57.4)	8 (57.1)	16 (61.5)	76 (30.8)	165 (36.3)
No	57 (66.3)	129 (73.7)	87 (82.1)	103 (68.7)	20 (51.3)	40 (42.6)	6 (42.9)	10 (38.5)	171 (69.2)	290 (63.7)
**Followed algorithm**										
Yes	72 (71.3)	149 (80.5)	82 (70.1)	125 (78.1)	24 (52.2)	90 (84.9)	7 (43.8)	16 (61.5)	186 (66.0)	389 (79.7)
No	29 (28.7)	36 (19.5)	35 (29.9)	35 (21.9)	22 (47.8)	16 (15.1)	9 (56.3)	10 (38.5)	96 (34.0)	99 (20.3)

* Variable not included in PT1

#### Assessing level of accuracy

Accuracy was expressed as a percent score for each tester/site, tester group and each PT [42] (Table 2). Accuracy ranged from 0% (no specimen correctly identified) to 100% (all five specimens correctly identified) for each PT. Mean scores were calculated and reported with 95% confidence intervals (CIs). The Kruskal-Wallis one-way analysis of variance (ANOVA) by ranks test was used to assess differences in accuracy between the tester groups. Post hoc multiple comparisons using a series of Mann-Whitney U tests with Bonferroni adjustment were used for pairwise comparisons to determine which groups were statistically significantly different from the other. Similar analyses were performed to assess whether differences in accuracy between PT1 and PT2 overall, and among sites that participated in both PT cycles were statistically significant.

**Table 2:** Proportion of tester groups who achieved a specified level of accuracy in PT1 and PT2.

	Lay counselors		Nurses		Laboratory personnel		Others		Overall	
	PT1	PT2	PT1	PT2	PT1	PT2	PT1	PT2	PT1	PT2
Accuracy (%)										
100	72.3	87.6	79.5	87.5	97.8	95.3	75.0	92.3	79.8	89.3
80	14.9	9.2	14.5	8.8	0.0	2.8	25.0	7.7	12.8	7.4
60	5.0	2.2	1.7	1.9	0.0	1.9	0.0	0.0	2.5	2.3
40	6.9	0.5	2.6	1.3	2.2	0.0	0.0	0.0	3.9	0.6
20	0.0	0.5	1.7	0.0	0.0	0.0	0.0	0.0	0.7	0.2
0	1.0	0.0	0.0	0.6	0.0	0.0	0.0	0.0	0.4	0.2
Mean score (%)	89.9	96.5	93.5	96.1	98.7	98.7	95.0	98.5	93.1	96.9
95%CI	85.9–93.5	94.9–97.9	90.5–96.0	94.1–97.9	95.5–100.0	97.3–99.7	90.6–98.9	95.9–100.0	91.2–94.9	96.1–97.8

Scores based on final status results. 100% = 5/5 tests correct; 80% = 4/5 tests correct; 60% = 3/5 tests correct; 40% = 2/5 tests correct; 20% = 1/5 tests correct; 0% = 0/5 tests correct

#### DTS panel tests results

The DTS coded as A1 to A5 (PT1) and B1 to B5 (PT2) were analyzed to obtain frequencies of correct, false (positive and negative) and discrepant results (Table 3), as well as to determine the level of agreement with the expected results. The reported false-negative, false-positive and indeterminate (discrepant) results were further investigated to determine which categories of testers reported them as such (Table 4).

**Table 3:** Expected and reported results for each DTS specimen in PT1 and PT2.

PT1					PT2				
DTS code	Expected results	Correct results	False results	Discrepant results	DTS code	Expected results	Correct results	False results	Discrepant results
A1	Negative	274	7	1	B1	Positive	455	27	4
A2	Positive	249	15	9	B2	Negative	476	4	4
A3	Positive	266	1	3	B3	Positive	485	1	1
A4	Positive	268	2	3	B4	Negative	473	4	3
A5	Negative	261	17	2	B5	Positive	474	5	5

Discrepant (or indeterminate) results: is when the screening and confirmatory test results for a sample are not concordant and therefore inconclusive. Samples A2 and B1 were weak positive specimens in PT1 and PT2 respectively. Row totals not all the same in PT1 and PT2 respectively due to missing results which were included as incorrect results.

**Table 4:** Distribution of reported false and indeterminate results by tester profession in PT1 and PT2.

PT1				PT2		
	False negative	False positive	Indeterminate	False negative	False positive	Indeterminate
	n (%)	n (%)	n (%)	n (%)	n (%)	n (%)
Lay counselors	9 (50.0)	10 (58.8)	12 (66.7)	20 (60.6)	3 (37.5)	10 (50.0)
Nurses	9 (50.0)	6 (35.3)	4 (22.2)	11 (33.3)	2 (25.0)	9 (45.0)
Lab personnel	0 (0.0)	0 (0.0)	0 (0.0)	0 (0.0)	3 (37.5)	1 (5.0)
Others	0 (0.0)	1 (5.9)	2 (11.1)	2 (6.1)	0 (0.0)	0 (0.0)
Total	18	17	18	33	8	20

#### Factors associated with accuracy in HIV rapid testing

Multiple linear regression analysis was used to examine factors associated with accuracy. The regression analyses were performed step-wise. Bivariate associations were presented first followed by the multivariate associations in four steps as seen in Table 5. Preliminary analyses were conducted to ensure no violation of the assumptions of normality, linearity and multicollinearity. No interactions were identified. All analyses were done first for the whole group then stratified by profession of tester. The dependent variable ‘accuracy’ was used as a continuous variable and was coded 0 to 5.

**Table 5:** Factors associated with accuracy in HIV rapid testing among all tester groups in PT2.

Univariate					Multivariate							
					Step 1		Step 2		Step 3		Step 4	
	n	Mean score (%)	beta	p-value	beta	p-value	beta	p-value	beta	p-value	beta	p-value
**Demographic factors**												
**Location of site**												
Rural	162	96.1	0		0		0		0		0	
Urban	326	97.3	0.059	0.194	0.059	0.194	0.060	0.270	0.049	0.406	0.042	0.514
**Training and supervision**												
**Training attended**												
Other/no training	150	96.5	0				0		0		0	
HIV rapid testing training	248	97.9	0.069	0.167			0.107	0.051	0.124	0.035	0.137	0.034
**Date last trained**												
> 1 year ago	249	96.6	0				0		0		0	
< 1 year ago	119	97.0	0.016	0.761			0.003	0.950	0.023	0.705	0.000	0.994
**Exam after training**												
No	46	96.7	0				0		0		0	
Yes	345	97.4	0.020	0.689			0.003	0.951	0.019	0.746	0.020	0.750
**Visited by trainer**												
No	109	96.5	0				0		0		0	
Yes	289	97.6	0.044	0.379			0.051	0.359	0.067	0.259	0.087	0.183
**HIV testing work experience**												
**No. of years of testing**												
< 1 year	61	95.7	0						0		0	
> 1 year	396	97.4	0.058	0.213					0.051	0.407	0.048	0.466
**No. of staff testing**												
0–5	216	97.2	0						0		0	
6–10	142	96.8	0.031	0.529					0.001	0.982	0.013	0.848
≥11	59	98.0	0.034	0.490					0.036	0.561	0.049	0.460
**Adherence to Procedures**												
**Perform IQC**												
No	149	96.8	0								0	
Yes	293	97.1	0.014	0.773							0.017	0.795
**Have a timer**												
No	290	96.6	0								0	
Yes	165	97.7	0.056	0.230							0.024	0.708
**Follow testing algorithm**												
No	99	94.3	0								0	
Yes	389	97.5	0.121	0.007							0.140	0.032
**R**^**2**^					0.004		0.017		0.025		0.050	

**Variables in the model****:** Step 1: Demographic factors. Step 2: HIV testing training attended. Step 3: HIV testing work experience. Step 4: Adherence to procedures. Results are standardized regression coefficients (beta) and explained variances (R^2^) from a multiple linear regression analysis

### Ethics

The Zambia National HIV testing Quality Assurance Program was reviewed and ethically approved by the Ministry of Health Review Board. No personal information was obtained from the testers. After the questionnaires were captured digitally, all identifying information from the sites was removed from the final dataset. All information was kept confidential.

## Results

### Participation and responses

A total of 550 sites received PT panels in PT1 and 282 responses (135 rural, 147 urban) were returned, giving a response rate of 51.3%. In PT2, a total of 488 responses (162 rural, 326 urban) were returned from the 680 targeted sites, giving an increased response rate of 71.8%. Further details of participation have been given elsewhere [40]. Of the 488 sites that participated in PT2, 180 sites also participated in PT1.

### General background and PT characteristics

The majority of testers were lay counselors and nurses, together accounting for 77.9% and 72.3% of the testers in PT1 and PT2 respectively (Table 1). Two-thirds of sites (66.8%) were located in urban areas in PT2 compared to 52.1% in PT1. Nearly two-thirds (62.3%) of the participants in PT2 reported having received the standard HIV rapid testing training, while others (mostly lay counselors and nurses) had received other trainings such as PMTCT training (12.8%) and psychosocial counseling training (19.8%). A small proportion (5.0%) had no formal HIV testing training at all, but reported having learnt to perform the HIV test from a supervisor or colleagues. Most testers had more than one year work experience in both PT1 (68.1%) and PT2 (86.7%). In PT2, most participants (91.4%) reported that it was easy to reconstitute the DTS, while a few (8.6%) found it difficult or were unable to reconstitute and were assisted by colleagues from the local laboratory. More than three-quarters of the participants (79.7%) followed the national HIV testing algorithm during PT2, with the most improvement seen among laboratory personnel at 84.9% from 52.2% in PT1. A similar pattern of characteristics was observed among sites that participated in both PT cycles.

### Accuracy and associated factors

The average overall accuracy level was 93.1% (95% CI: 91.2–94.9), range: 89.9%–98.7% in PT1 and 96.9% (95% CI: 96.1–97.8), range: 96.1%–98.7% in PT2 (Table 2). A significant upward difference was revealed between PT1 and PT2 (U = 62089, p = 0.000). Further, among sites that participated in both PT cycles, a significant upward difference was revealed from PT1 to PT2 (U = 12155, p = 0.005), with overall accuracy levels of 91.4% (95%CI: 88.2–94.4) and 96.7% (95%CI: 95.1–98.1) respectively. Comparing the two exercises, an improvement in accuracy level was seen among all non-laboratory tester groups, i.e. lay counselors (96.5% from 89.9%), nurses (96.1% from 93.5%) and others (98.5% from 95.0%), while performance remained stable among laboratory personnel (98.7% vs. 98.7%). Among all the testers, 79.8% and 89.3% attained 100% accuracy scores in PT1 and PT2 respectively, with laboratory personnel obtaining the highest scores in both PT cycles.

A statistical difference in accuracy at p<0.05 level was revealed across the tester groups (χ^2^ = 12.75, p = 0.005) in PT1. Pairwise comparisons revealed a statistical difference in accuracy between lay counselors and laboratory personnel (U = 1739, p = 0.000); nurses and laboratory personnel (U = 2206, p = 0.004); and laboratory personnel and others (U = 286, p = 0.005). There was no statistical difference in accuracy between lay counselors and nurses (U = 5433, p = 0.170); lay counselors and others (U = 760, p = 0.626); or nurses and others (U = 908, p = 0.785). There was no statistical difference in accuracy across the tester groups (χ^2^ = 5.38, p = 0.146) in PT2.

Of the 1410 results in PT1 and 2440 results in PT2, 1318 (93.4%) in PT1 and 2362 (96.8%) in PT2 were in agreement with expected results. Of all the false-negative results, 15 and 27 were reported for the weak positive specimens in PT1 and PT2 respectively (Table 3). False-negative results were reported mostly by lay counselors and nurses in both PT1 and PT2. False-positive and indeterminate results were reported by lay counselors and nurses in both PT1 and PT2, by others only in PT1 and by laboratory personnel only in PT2 (Table 4).

Accuracy did not differ statistically by location of the test site (Table 5). The single most important indicator of accuracy was “following the national HIV testing algorithm”, p = 0.032. Having received the standard HIV rapid testing training was also found to somewhat increase accuracy compared to other training options. None of the other indicators were significantly associated with accuracy. Stratification by tester group revealed a similar pattern of associations across all tester groups. The explained variance (R^2^) was generally low.

## Discussion

The majority of the testers were lay counselors and nurses in both PT rounds. The overall accuracy level was 93.1% and 96.9% (p = 0.000) in 2009 and 2010 respectively. Further, among sites that participated in both PT cycles, accuracy level was 91.4% and 96.7% (p = 0.005) in PT1 and PT2 respectively. Differences in accuracy were seen between tester groups in 2009, with laboratory personnel being more accurate than lay counselors, nurses and others (p = 0.005), while in 2010, no statistically significant differences were seen. Supposing that the 2010 results represent the national accuracy level and that at least 3 million tests were performed [43], nearly 100,000 individuals would have received an incorrect HIV test result. The seemingly small error rate therefore has substantial implications for many individuals. Having received the standard HIV rapid testing training and adherence to the national HIV testing algorithm were positively associated with testers’ accuracy.

Lay counselors and nurses together constituted the largest group of testers, indicating a rapid implementation of task-shifting in HIV testing services [44] and hence a need to concentrate efforts in these groups to ensure higher levels of accuracy. In the first round, laboratory personnel were substantially more accurate than the non-laboratory personnel. However, no significant variation in accuracy between the tester groups was seen in the second round due to a great improvement among all non-laboratory tester groups and particularly among lay counselors. We see this as an indication that there is a great potential for these groups to attain higher accuracy levels and perform testing as well as laboratory personnel. One possible explanation as previously suggested is that lay counselors may be more receptive to learning new procedures that are outside their routine occupations [11]. Although laboratory personnel showed a stable performance in both PT cycles, there is a need for continuous training to improve competencies [12,25]. Though a specimen reconstitution step that is not normally required in routine testing was introduced, the fact that it was found to be easy by nearly all the testers showed that they were comfortable with DTS as PT material for EQA [12].

A significant improvement in performance was revealed from PT1 to PT2 overall, and among sites that participated in both PT cycles. Coupled with the individual tester group results, these findings suggest that PT implementation has a positive impact on performance. Possible reasons for this improvement could have been the site supervisory visits (SSVs) by the NRL experts [45], as well as improved adherence to testing procedures, which could be directly linked to increased training. These findings suggest the need for EQA programs in collaboration with their MOHs to prioritize investment in SSVs as well as training of testers, regardless of setting or profession, for enhanced performance.

Our study revealed some difficulties in interpretation of results in both PT rounds, with the occurrence of false-negative, false-positive and indeterminate interpretations. The problems tended to occur more among lay counselors and nurses than laboratory personnel and others. According to the national HIV testing algorithm, specimens that give discrepant results between the screening and confirmatory tests should be subjected to the tie-breaker test. On the other hand, specimens that test negative on the screening test are not tested further, thus false-negative interpretations are not defined in the testing algorithm. The occurrence of false-negative interpretations was of concern, and highlights the need to further investigate the ability of testers to correctly interpret test results and the potential need for further training [19]. We cannot exclude the possibility that the occurrence of false-positives could have arisen from cross-contamination due to poor handling techniques during the processing of the DTS, i.e. at the point of reconstitution since only one transfer pipette was provided per PT package, or at the point of testing. These findings support previous publications suggesting that even though HIV rapid tests may be easy to perform, they may be fraught with difficulties in interpretation of results particularly if testers have limited training and experience [14,19]. Further, our findings confirm previous observations that non-laboratory personnel may have more difficulties of this kind [28,29]. Based on our findings, a review of the current training and the development of a curriculum that is tailor-made to profession of testers may be helpful. The training duration could be increased to include more practical sessions. A previous home based VCT study in rural Zambia found that with more comprehensive training and hands-on practical experience, lay counselors were able to perform testing proficiently and achieved a 100% accuracy score during a PT exercise [10].

We assumed that the level or type of training would affect testing accuracy [14,19]. Our results revealed accuracy to be associated only with exposure to HIV rapid testing training as a focused training, as compared to having received other trainings. The standard national HIV rapid testing training is a three-day training focused on principles of HIV rapid testing and practical sessions for hands-on experience [12,39]. Other training programs have various components included in their training curricula, with some components such as counseling being more emphasized than testing [40]. Our findings suggest offering all testers the standard HIV rapid testing training, regardless of any other training they may have previously received. In addition, reports that were given by the testers during the PT exercises revealed a multiplicity of trainers (about 40 organizations) that had provided HIV rapid testing training of varied content and duration ranging from 2 hours to 6 months. This shows the need for the establishment of a central coordinating body e.g. the NRL or equivalent institution by the MOH to ensure training is standard for all testers [12].

In this investigation, having more than one year work experience was not associated with accuracy, contrary to previous publications [12,19]. Similarly, the data did not support an association between accuracy and having more numbers of skilled staff performing HIV testing. Inadequate numbers of skilled staff in a facility could translate into a higher workload for the few available staff. This has been found to have a negative impact on quality of service, particularly in public institutions [46–48]. Many HIV testing facilities report high staff turnover and attrition, with rural public facilities having the lowest numbers of skilled staff as compared to urban facilities [46,49]. Further, many rural sites are located in remote areas which may pose a challenge to quality service provision with regards to easy access to test kits and reagents, training as well as external supervision, communication and networking with other testing sites at regional or national level [24]. Strategies for staff retention and improved service provision conditions need to be made a central issue in health policy [46].

Adherence to the national HIV testing algorithm was found to be positively associated with accuracy. An algorithm involves the use of categories of tests in a particular sequence which have been selected and validated through an evaluation process [12,50]. Thus an alteration in sequence could have a negative impact on test sensitivity and specificity. Non-adherence to the national testing algorithm was defined as performing parallel testing, confirmatory testing on specimens that are non-reactive on the screening test, reporting positive results based on one test (screening test) or confirming a reactive specimen with the wrong test (tie-breaker). Though parallel testing and confirming non-reactive specimens did not have a negative impact on accuracy, such practices in a serial testing strategy may lead to wastage of test kits and increase the cost of reagents [12]. However, reporting positive results based on one test and confirming a reactive specimen with the wrong test were of concern. The reasons for such practices could have been limited training and understanding, or non-availability of confirmatory test kits.

A global concern on the use of tie-breakers in routine testing has been raised. Tie-breakers have been found to not always resolve the HIV status with accuracy [12]. Some studies have found that due to antibody cross-reactivity, i.e. the production of antibodies by various foreign antigens and infectious agents that non-specifically cross-react with antigens in the test kits, the use of a tie-breaker has sometimes led to false-positive diagnosis. This has led to individuals being wrongly enrolled on ART programs, as well as devastating individual consequences. Some studies have suggested re-testing of individuals already on ART programs, as well as samples testing positive on the tie-breaker with confirmatory tests such as Western blot or PCR. A few studies have also indicated a need for ART and prevention programs to plan for quality assurance in HIV testing [51,52,53]. Other studies however have found high accuracy in HIV testing using an algorithm that includes a tie-breaker [54]. The outcome thus depends on the test kit that is selected to be used as a tie-breaker. In some countries, a blood specimen is collected from the individual with discordant results and sent to a facility capable of performing further testing to confirm the HIV status. But often, rather than using a tie-breaker, clients are re-tested after 4 weeks to resolve the HIV status. This approach has been found to be more likely to provide a correct diagnosis, though clients may not return for follow-up testing. Appropriate counseling may help ensure a return visit [12].

Selection bias might have been one of the possible biases in this study. The sample was not a random sample of all the sites in the country. However, sites were selected to represent all types and levels of facilities in both rural and urban areas, i.e. public, private and mission. Therefore, though only a few sites (30%) of the total 1689 registered sites countrywide were reached by the program by PT2, the findings are likely to reasonably well portray the national situation. However, the selection of rural sites could to some extent have been biased towards ease of access, thereby possibly leaving out those sites most challenged in provision of quality service and thus we are likely to have over-estimated rural accuracy. Further, non-participation, which was due to hard-to-reach sites not receiving the PT materials or reporting results to the NRL on time, might have been another possible source of bias. This type of non-response might have also led to over-estimation of accuracy. Non-response was substantially lower in PT2 than in PT1, with the involvement of the national courier and MOH partners in the distribution of the PT materials and the return of the results to the NRL for analysis. Therefore generalization of rural or remote area accuracy may be restricted to the selected sites and from which reports were received. Self-reports of training, experience and adherence to procedures are subject to recall and reporting biases which might to some extent have diluted the estimated associations. PT involves only a few specimens and the test results may not represent routine test performance. This may partly be due to the greater care that is taken by testers in handling PT specimens [50]. Further, the person assigned to test the specimens may be the most experienced at the site. Accordingly, the direction of these biases would be an over-estimation of accuracy, but the magnitude was not possible to estimate.

A particular limitation in this study was that each PT round differs from the previous with the addition of new sites and mostly with different testers participating due to high staff attrition. A more optimal design would have been a cohort design to observe the same testers and sites and measure accuracy over a period of time, thus the impact of quality assurance systems. The use of retrospective data has its limitations in that the data was collected for routine program purposes and not particularly for this study and therefore we were restricted to the available data. In addition, the areas of missing data for some of the variables could to some extent have biased the associations. Another limitation is that due to limited resources, PT exercises are currently conducted in a few sites of the total sites countrywide, and are conducted only once a year thereby limiting coverage and closer monitoring of performance. Possible suggestions to reduce some of the limitations may include the use of a decentralized approach in combination with the MOH partners and other local organizations that could be trained to run the PT program and provide coverage to sites in specific geographical areas. Provincial reference laboratories could be set up to oversee and compile data for overall analysis and coordination by the NRL [12].

## Conclusions

The study showed that there was an improvement in tester groups and overall accuracy level in HIV rapid testing from the first to the second exercise. Further improvement is urgently needed, however, and the national HIV PT system could be an important tool in this regard which will need to be strengthened and given higher priority in terms of investment. The findings showed that task-shifting in HIV testing services has taken effect in Zambia, with lay counselors and nurses constituting the majority of testers. The type of training testers received and adherence to testing procedures appeared critical in achieving higher levels of accuracy. A regulatory framework for training is needed to ensure central coordination of training that is standard.

## References

[1] Merson MH, O’Malley J, Serwadda D, Apisuk C. The history and challenge of HIV prevention. The lancet. 2007; 372(9637):475–88. 10.1016/S0140-6736(08)60884-3 18687461

[2] UNAIDS. UNAIDS Report on the global AIDS epidemic. Geneva: UNAIDS; 2013.

[3] UNAIDS. HIV Voluntary Counseling and Testing: A gateway to prevention and care UNAIDS Best Practice collection, ed. Geneva: UNAIDS; 2002.

[4] WHO, UNAIDS. Policy statement on HIV testing. Geneva: WHO; 2004.

[5] Babalola S. Readiness for testing among young people in northern Nigeria: the roles of social norm and perceived stigma. AIDS Behav. 2007; 11(5):759–69. 17191141 10.1007/s10461-006-9189-0

[6] Fylkesnes K, Haworth A, Rosensvard C, Kwapa PM. HIV counseling and testing: over-emphasizing high acceptance rate a threat in confidentiality and the right to know. AIDS. 1999; 13(17):2469–74. 10597789 10.1097/00002030-199912030-00019

[7] Jurgensen M, Tuba M, Fylkesnes K, Blystad A. The burden of knowing: balancing benefits and barriers in HIV testing decisions. A qualitative study from Zambia. BMC Health Serv Res. 2012; 12:2 10.1186/1472-6963-12-2 22222028 PMC3268706

[8] WHO, UNAIDS. Guidance on provider-initiated HIV testing and counseling in health facilities. Geneva: WHO; 2007.

[9] Wanyenze RK, Nawavvu C, Namale AS, Mayanja B, Bunnell R, Abang B, et al Acceptability of routine HIV counseling and testing and HIV seroprevalence in Ugandan hospitals. Bull World Health Organ. 2008; 86(4):302–9. 18438519 10.2471/BLT.07.042580 PMC2647415

[10] Fylkesnes K, Sandoy IF, Jurgensen M, Chipimo PJ, Mwangala S, Michelo C. Strong effects of home-based voluntary HIV counseling and testing on acceptance and equity: a cluster randomized trial in Zambia. Soc Sci Med. 2013; 86:9–16. 10.1016/j.socscimed.2013.02.036 23608089

[11] Yao K, Wafula W, Bile EC, Cheignsong R, Howard S, Demby A, et al Ensuring the quality of HIV rapid testing in resource-poor countries using a systematic approach to training. Am J Clin Pathol. 2010; 134(4):568–72. 10.1309/AJCPOPXR8MNTZ5PY 20855637

[12] Parekh BS, Kalou MB, Alemnji G, Ou CY, Gershy-Damet GM, Nkengasong JN. Scaling up HIV rapid testing in developing countries: comprehensive approach for implementing quality assurance. Am J Clin Pathol. 2010; 134(4):573–84. 10.1309/AJCPTDIMFR00IKYX 20855638

[13] Schalla WO, Hearn TL, Taylor RN, Eavenson E, Valdiserri RO, Essien JD. CDC’s model performance evaluation program: assessment of the quality of laboratory performance for HIV-1 antibody testing. Public Health Rep. 1990; 105(2):167–71. 2157234 PMC1580063

[14] Chiu YH, Ong J, Walker S, Kumalawati J, Gartinah T, McPhee DA, et al Photographed rapid HIV tests results pilot novel quality assessment and training schemes. PLoS One. 2011; 6(3):e18294 10.1371/journal.pone.0018294 21483842 PMC3069085

[15] Koblavi-Dème S, Maurice C, Yavo S, Sibailly TS, N’guessan K, Kamelan-Tano Y, et al Sensitivity and specificity of HIV rapid serologic assays and testing algorithms in an antenatal clinic in Abidjan, Ivory Coast. J Clin Microbiol. 2001; 39:1808–12. 11325995 10.1128/JCM.39.5.1808-1812.2001 PMC88030

[16] WHO. HIV assays: operational characteristics Report 16. Rapid assays. Geneva: WHO; 2009 Available: http://www.who.int/diagnostics_laboratory/publications/Report16_final.pdf. Accessed 10 Nov 2012.

[17] WHO. Treat, train, retain: the AIDS and health workforce plan: report on the consultation on AIDS and human resources for health. Geneva: WHO; 2006 Available: http://www.who.int/hiv/pub/meetingreports/TTRmeetingreport2.pdf. Accessed 9 Oct 2012.

[18] WHO: Task shifting: rational redistribution of tasks among workforce teams: global recommendations and guidelines. Geneva: WHO; 2008 Available: http://www.who.int/healthsystems/TTR-TaskShifting.pdf. Accessed 12 Oct 2012.

[19] Learmonth KM, McPhee DA, Jardine DK, Walker SK, Aye TT, Dax EM. Assessing proficiency of interpretation of rapid human immunodeficiency virus assays in nonlaboratory settings: ensuring quality of testing. J Clin Microbiol. 2008; 46(5):1692–7. 10.1128/JCM.01761-07 18353938 PMC2395071

[20] Zimbabwe Quality Assurance Program (ZINQAP). Harare: ZINQAP; 2012. Available: http://www.zinqap.org.zw. Accessed 15 Nov 2012.

[21] Jiang Y, Qiu M, Zhang G, Xing W, Xiao Y, Pan P, et al Quality assurance in the HIV/AIDS laboratory network of China. Int J Epidemiol. 2010; 39(suppl 2):ii72–8. 10.1093/ije/dyq224 21113040 PMC2992624

[22] Sushi KM, Gopal T, Jacob SM, Arumugam G, Durairaj A. External quality assessment scheme in a national reference laboratory for HIV testing in South India. WJA. 2012; 2:222–5.

[23] Chang D, Learmonth K, Dax EM. HIV testing in 2006: issues and methods. Expert Rev Anti Infect Ther. 2006; 4(4):565–82. 17009937 10.1586/14787210.4.4.565

[24] Alemnji G, Nkengasong JN, Parekh BS. HIV testing in developing countries: What is required? Indian J Med Res. 2011; 134(6):779–86. 10.4103/0971-5916.92625 22310813 PMC3284089

[25] Ministry of Health, Republic of Zambia. National Quality Assurance strategy for HIV counseling and testing. Lusaka: Ministry of Health; 2007.

[26] Stull TM, Hearn TL, Hancock JS, Handsfield JH, Collins CL. Variation in Proficiency testing performance by testing site. JAMA. 1998; 297(6):463–7. 10.1001/jama.279.6.463 9466641

[27] WHO. Guidelines for assuring the accuracy and reliability of HIV rapid testing: applying a quality system approach. Geneva: WHO; 2005.

[28] Kanal K, Chou TL, Sovann L, Morikawa Y, Mukoyama Y, Kakimoto K. Evaluation of the proficiency of trained non-laboratory health staffs and laboratory technicians using a rapid and simple HIV antibody test. AIDS Res Ther. 2005; 2:5 15907202 10.1186/1742-6405-2-5 PMC1156864

[29] Martin R, Hearn TL, Ridderhof JC, Demby A. Implementation of a quality systems approach for laboratory practice in resource-constrained countries. AIDS. 2005; 19(suppl 2):S59–65. 15930842 10.1097/01.aids.0000172878.20628.a8

[30] WHO, WHO/EHT, UNAIDS. HIV assays: operational characteristics (phase 1). Report 12 Simple/rapid tests, whole blood specimens. Geneva: WHO; 2002 Available: http://www.who.int/diagnostics_laboratory/publications/hiv_assays_rep_12.pdf. Accessed 10 Nov 2012.

[31] Sandoy IF, Kvale G, Michelo C, Fylkesnes K. Antenatal clinic-based HIV prevalence in Zambia: declining trends but sharp local contrasts in young women. Trop Med Int Health. 2006; 11(6):917–28. 16772014 10.1111/j.1365-3156.2006.01629.x

[32] Kayeyi N, Fylkesnes K, Michelo C, Makasa M, Sandoy I. Decline in HIV prevalence among young women in Zambia: National-level estimates of trends mask geographical and socio-demographic differences. PLoS One. 2012; 7(4):e33652 10.1371/journal.pone.0033652 22496759 PMC3319534

[33] Makasa M, Fylkesnes K, Michelo C, Kayeyi N, Chirwa B, Sandoy I. Declining syphilis trends in concurrence with HIV declines among pregnant women in Zambia: observations over 14 years of national surveillance. Sex Transm Dis. 2012; 39(3):173–81. 10.1097/OLQ.0b013e31823b23a4 22337102

[34] Michelo C, Sandoy IF, Fylkesnes K. Marked HIV prevalence declines in higher educated young people: evidence from population based surveys (1995–2003) in Zambia. AIDS. 2006; 20:1031–8. 16603856 10.1097/01.aids.0000222076.91114.95

[35] Sandoy IF, Michelo C, Siziya S, Fylkesnes K. Associations between sexual behavior change among young people and decline in HIV prevalence in Zambia. BMC Public Health. 2007; 7:60 17448256 10.1186/1471-2458-7-60 PMC1868719

[36] WHO, UNAIDS, UNICEF. Global HIV/AIDS response, epidemic update and health sector progress towards universal access. Geneva: WHO; 2011.

[37] Central Statistical Office (CSO), Ministry of Health (MOH), Tropical Diseases Research Centre (TDRC), University of Zambia (UNZA), Macro International Inc. Zambia Demographic and Health Survey 2007. Calverton, Maryland, USA: CSO and Macro International Inc; 2009.

[38] Central Statistical Office, Ministry Of Health, National HIV/AIDS/STI/TB council, University of Zambia, MEASURE Evaluation. Zambia Sexual Behavior Survey 2009. In. Lusaka: CSO, MOH, NAC, UNZA, MEASURE Evaluation; 2010.

[39] Ministry of Health, National HIV/AIDS/STI/TB council. National HIV rapid test training curriculum. Lusaka: Ministry of Health; 2007.

[40] Ministry of Health (MOH), Virology Laboratory. 1st proficiency testing trial preliminary draft report. Lusaka: Virology Laboratory; 2009.

[41] Parekh B, Anyanwu J, Patel H, Downer M, Kalou M, Gichimu C, et al Dried tube specimens: A simple and cost-effective method for preparation of HIV proficiency testing panels and quality control materials for use in resource–limited settings. J Virol Methods. 2009; 163:295–300. 10.1016/j.jviromet.2009.10.013 19878697

[42] UNAIDS. Guidelines for organizing national external quality assessment schemes for HIV serological testing. UNAIDS; 1996.

[43] Sikasote J, Chanda S, Nichodemus W. Zambia: National Quantification of HIV test kits (2009–2015). Lusaka, Zambia: USAID| Deliver Project; 2008.

[44] Sanjana P, Torpey K, Schwarzwalder A, Simumba C, Kasonde P, Nyirenda L, et al Task-shifting HIV counseling and testing services in Zambia: the role of lay counselors. Hum Resour Health. 2009; 7:44 10.1186/1478-4491-7-44 19480710 PMC2692981

[45] Bukve T, Røraas T, Riksheim BO, Christensen NG, Sandberg S. Point-of-care urine albumin in general practice offices: effect of participation in an external quality assurance scheme. Clin Chem Lab Med. 2015; 53(1):45–51. 10.1515/cclm-2014-0483 25153401

[46] World Health Organization. Scaling up HIV testing and counseling services: A toolkit for programme managers. Geneva: WHO; 2005.

[47] Mbilinyi D, Daniel ML, Lie GT. Health worker motivation in the context of HIV care and treatment challenges in Mbeya region, Tanzania: a qualitative study. BMC Health Serv Res. 2011; 11:266 10.1186/1472-6963-11-266 21992700 PMC3214153

[48] Kruse GR, Chapula BT, Ikeda S, Nkoma M, Quiterio N, Pankratz D, et al Burnout and use of HIV testing services among health care workers in Lusaka district, Zambia: A cross-sectional study. Hum Resour Health. 2009; 7:55 10.1186/1478-4491-7-55 19594917 PMC2714832

[49] Ministry of Health. ACTION PLAN 2011. Lusaka, Zambia: Ministry of Health; 2011:6–16.

[50] World Health Organization Regional Office for Africa, Centres for Disease Control and Prevention, Association of Public Health laboratories. Guidelines for appropriate evaluations of HIV testing technologies in Africa. Atlanta: Centres for Disease Control and Prevention; 2003.

[51] Ndase P, Celum C, Kidoguchi L, Ronald A, Fife KH, Bukusi E, et al Frequency of false positive rapid HIV serologic tests in African men and women receiving PrEP for HIV prevention: Implications for programmatic roll-out of biomedical interventions. PLoS One. 2015; 10(4): e0123005 10.1371/journal.pone.0123005 25885664 PMC4401675

[52] Shanks L, Klarkowski D, O'Brien DP. False positive HIV diagnoses in resource limited settings: operational lessons learned for HIV programmes. PLoS One. 2013; 8(3):e59906 10.1371/journal.pone.0059906 23527284 PMC3603939

[53] Shanks L, Siddiqui M, Kliescikova J, Pearce N, Ariti C, Muluneh L, et al Evaluation of HIV testing algorithms in Ethiopia: the role of the tie-breaker algorithm and weakly reacting test lines in contributing to a high rate of false positive HIV diagnoses. BMC Infect Dis. 2015; 15:39 10.1186/s12879-015-0769-3 25645240 PMC4331460

[54] Molesworth AM, Ndhlovu R, Banda E, Saul J, Ngwira B, Glynn JR, et al High accuracy of home-based community rapid HIV testing in rural Malawi. J Acquir Immune Defic Syndr. 2010; 55(5):625–30. 10.1097/QAI.0b013e3181f98628 21934554 PMC3248920

